# Organ-on-Chip Technology: Current State and Future Developments

**DOI:** 10.3390/genes8100266

**Published:** 2017-10-11

**Authors:** Rimantas Kodzius, Frank Schulze, Xinghua Gao, Marlon R. Schneider

**Affiliations:** 1 German Federal Institute for Risk Assessment (BfR), German Centre for the Protection of Laboratory Animals (Bf3R), Berlin 10589, Germany; Frank.Schulze@bfr.bund.de (F.S.); marlon.schneider@bfr.bund.de (M.R.S.); 2 iSmart, Materials Genome Institute, Shanghai University (SHU), Shanghai 201800, China; gaoxinghua@t.shu.edu.cn; 3 Mathematics and Natural Sciences Department, The American University of Iraq, Sulaimani, Sulaymaniyah 46001, Iraq

In the early days of pharmacy, the development of new drugs was frequently achieved by restless chemists who worked solitarily, day by day for years. As an example, it took two intensive years of work for Friedrich Sertürner to isolate and purify the pain medicine morphine, a drug derived from a mixture of substances known as opium in the year 1804 [1]. In those days, chemists tested the newly isolated or synthesized formulations by giving these to animals found in the vicinity of their laboratory, whether these were frogs, mice, or dogs. The dosing was adjusted just to be “sure” not to cause severe toxicity or death when scientists applied these newly synthesized chemicals on themselves or volunteers. Over the last two hundred years, science progressed enormously. Classical pharmacology can now utilize cells in culture for the screening of various small molecules (potential drugs) so that their therapeutic value can be identified. New drugs are now also searched or assessed computationally (in silico). Further, the human genome has been sequenced and is thus known in depth, a prerequisite for computational approaches that aim to recreate human physiology; the objective is to find possible candidates with therapeutic potential, and for that, large libraries of compounds are screened. In this in silico search, some important parameters are included to ensure better drug affinity, bioavailability, metabolic stability, and fewer side effects (selectivity). After the identification of a suitable compound, the new chemical formulation undergoes rigid pre-clinical research on microorganisms, eukaryotic cells in culture and living animals. The new chemical entity will be tested for its toxicity, safety, pharmacokinetics, and metabolism. Only then, after successful demonstration of the required effect without causing toxicity, can the regulatory approval be given to move into clinical trials (Phase I, II, III, and IV in humans). Two centuries ago, it took two years alone for the isolation of an active substance by one chemist. Nowadays, pharmaceutical companies seek to accelerate drug discovery. However, the development of new drugs still takes a minimum of two years and is now performed by groups of scientists and associated with costs of hundreds of millions of dollars, and in extreme cases over a billion of dollars [2]. Yet, the discovery of new pharmacologic active compounds is not performed by screening one substance after the other but in parallel and with the help of advanced in silico and in vitro methods (i.e., alternative methods) that were far from being available 200 years ago and, most notably, without risking lives.

These alternative methods have the potential not only to precede animal testing and add information but also to replace animal experiments completely. The many established cell lines derived from cancer tissue or by directed genetic modification contribute to straightforward screening approaches. Furthermore, due to advances in cell culture techniques and close collaboration with clinicians, more and more work has been done on primary cells obtained from patients or healthy donors. While conventional cell cultures use homogeneous cell populations cultivated in monolayer under static conditions, the development of Organs-on-a-Chip (OoCs) will enable more sophisticated systems that mimic organs, tissues, or whole organisms.

To reproduce physiological conditions, a whole battery of parameters needs to be considered when maintaining cells in culture. Temperature, acidity, air composition (O_2_, CO_2_), and even mechanical forces—these are all parameters that can be controlled in smart wells or microchips. The development of silicone, plastic, and glass chips and related microfluidic control systems push the boundaries of science and open up unseen potentials. The on-chip control of environmental parameters, the precise application of drug molecules, and precise readouts are now possible and help to cultivate and analyze cells and tissues in a more physiological environment. OoC technology is being employed by a growing number of laboratories worldwide. A variety of chip designs allows for the simulation of virtually every tissue in the human body, including the heart, lung, kidney, bone, and gut, among many others. The combination of various organ models on-chip facilitates a better understanding of a drugs’ action in a precisely controlled physiological environment. Further, multi-organ systems also take intermediate steps such as metabolism or barrier-transfer into account. The on-chip comparison of animal and human tissues will also help to understand species-specific differences and thus improve estimates on drug action in humans. Ultimately, a human-like system on a chip may be created, in which drug metabolism and effects on other organs can be determined. With the development of personalized medicine, tissues and cells from heterogenic origins can be used on OoCs; we can thus expect the rising need for ethical clarification and for laying down the regulations to use these cells and tissues.

In this special issue of *Genes*, we gathered research and review articles that cover new technological developments in the OoC field. These include novel engineering approaches in OoC manufacturing, new developments in sensor technology, and the use of OoC systems in the discovery of new drugs and their efficient delivery. Using primary cells facilitates the physiologic modeling of healthy and diseased tissue while taking the heterogeneity in genetic backgrounds of humans into account. Yet, utilizing this material is connected to ethical and legal issues that will also be addressed. Furthermore, this special issue includes reviews that focus on specific OoC systems such as lung and bone.

We can foresee a great future and a variety of applications for OoC. Drug testing, toxicity studies, pharmacology, cosmetics industry, cancer research, and material testing for implants are just a few of the fields that could benefit from this technology. Of course, there is a long way to go before reaching these objectives. Microelectronics and microfluidic control systems are being constantly improved and developed further. Major challenges for the coming years include a better understanding of cell–material interactions and the exact recreation of physiological parameters of the tissue of interest. Sharing the advances in this special issue will help to develop the OoC field. We thank the authors, reviewers, and editors for their commitment and for sharing their knowledge in the form of reviews and original data articles.

## Brief Guest Editors’ biographies:

Prof. **Rimantas Kodzius** studied chemistry and biochemistry, and graduated with a Ph.D. in Molecular Medicine at Paris Lodron University of Salzburg in Austria in 2002. Dr. Kodzius worked in famous universities and institutes in various other countries during his career. He has been to Austria, Germany, Switzerland, Sweden, Japan, Australia, Singapore, Hong Kong, Saudi Arabia, China, and Iraq. Dr. Kodzius is a founding member of the King Abdullah University of Science & Technology (KAUST) in Saudi Arabia. The prestigious journal *Science* published an interview in 2017 where Dr. Kodzius was designated as “the most migratory scientist in the world”.

Dr. Kodzius’ research interests encompass molecular medicine, microfluidic-methods, and bioprospecting. Dr. Kodzius also provides the technical expertise for multiple granting agencies such as EuroStars (Programme for Small and Medium-sized Enterprises), for the European Commission Research Executive Agency (ECR EA and as a Marie Evaluation expert of Marie Sklodowska-Curie Individual Fellowships), and for the EU parliament as a scientific adviser.

Dr. Kodzius has multiple publications in high-impact factor journals such as *Science*, *Nature Genetics*, *Genome Biology*, and *Nature Methods*.

Dr.-Ing. **Frank Schulze** is currently working as a post-doctoral researcher at the German Centre for the Protection of Laboratory Animals (Bf3R) that is embedded in the German Federal Institute for Risk Assessment (BfR). He received his Ph.D. in Biotechnology from the Technical University of Berlin in 2015. Dr.-Ing Schulze has worked in the field of stem cells, nanoparticle–cell interactions, and bone biology. His current research interests encompass tissue engineering and microfluidic organ-on-chip systems as alternative approaches to animal testing.

Dr. **Xinghua Gao** is currently an assistant professor in Materials Genome Institute, Shanghai University. She received a Ph.D. degree in 2013 from Dalian Institute of Chemical Physics (DICP), Chinese Academy of Sciences. She worked as a post-doctoral researcher in the Biomedical Research Institute, Shenzhen Peking University—The Hong Kong University of Science and Technology Medical Center from 2013 to 2015. Her research interests are mainly focused on the integration of microfluidics and biomimetic microsystem capable of investigating cancer biology, intelligent biomaterials, and tissue engineering.

Prof. **Marlon R. Schneider** graduated in Veterinary Medicine in 1995 and obtained a M.Sc. degree in 1997 from the Universidade Federal do Rio Grande do Sul in Porto Alegre, Brazil. From 1998 to 2001, he was the recipient of a fellowship from the German Academic Exchange Service (DAAD) and obtained a Dr. med. vet. degree from the Ludwig-Maximilians-University (LMU) Munich in 2001. After postdoctoral training at the Department of Genetics and Development, Columbia University, New York City (2001-2002), he was a research associate and then a group leader at the Gene Center, LMU Munich. He obtained a *venia legendi* for Genetics and Biotechnology (2010) and for Physiology (2015) from the LMU Munich. He is an editorial board member of *Experimental Dermatology* and an academic editor of *PLOS ONE*, and has served as a reviewer for numerous scientific journals and granting agencies.

Since 2016, he has led the Unit ZEBET – Alternative Methods to Animal Experiments at the German Centre for the Protection of Laboratory Animals (Bf3R), where he has focused on the development of alternative methods to animal experiments according to the 3R principle (reduce, replace, refine).
